# Rift Valley Fever Epizootic, Rwanda, 2022

**DOI:** 10.3201/eid3010.240264

**Published:** 2024-10

**Authors:** Eric Remera, Edson Rwagasore, Olivier Nsekuye, Muhammed Semakula, Misbah Gashegu, Robert Rutayisire, Leandre Ishema, Clarisse Musanabaganwa, Yvan Butera, Sabin Nsanzimana, Claude M. Muvunyi, Ayman Ahmed

**Affiliations:** Rwanda Biomedical Center, Kigali, Rwanda (E. Remera, E. Rwagasore, O. Nsekuye, M. Gashegu, R. Rutayisire, L. Ishema, C. Musanabaganwa, C.M. Muvunyi, A. Ahmed);; Ministry of Health, Kigali (M. Semakula, Y. Butera, S. Nsanzimana);; University of Khartoum, Khartoum, Sudan (A. Ahmed)

**Keywords:** Rift Valley fever, Rift Valley fever virus, zoonotic arboviral diseases, food insecurity, emerging infectious diseases, pandemic preparedness and prevention, multisectoral transdisciplinary One Health, Global Health Security, zoonoses, viruses, East Africa

## Abstract

A Rift Valley fever epizootic affected livestock in Rwanda during March–October 2022. We confirmed 3,112 infections with the virus, including 1,342 cases, 1,254 abortions, and 516 deaths among cattle, goats, and sheep. We recommend a One Health strategy for investigations and response to protect animal and human health.

Rift Valley fever (RVF) is a zoonotic arthropod-borne viral (arboviral) disease that affects a wide range of susceptible hosts, including livestock (cattle, sheep, goats), wildlife, and humans (*1*). It is caused by RVF virus (RVFV), which is mainly transmitted by *Aedes* and *Culex* mosquito vectors. Infection can also be acquired through close contact with infected animals or consumption of their infected products (e.g., raw milk, uncooked meat). In addition, vertical and sexual transmission of RVFV among humans, animals, and disease vectors has been documented (*2*). The disease affects health security and socioeconomics, leading to food insecurity and poverty, mainly among animal-resource–dependent communities (*3*). RVFV transmission is influenced by climate, increased mobility, and contact between infected and susceptible hosts (e.g., humans, animals, and vectors) (*4*,*5*). Emergence of RFV epidemics, epizootics, and outbreaks is associated with extreme whether events, such as heavy rains and flooding (*2*,*6*).

Apart from Saudi Arabia and Yemen, RVF is confined to Africa; countries in East Africa (e.g., Sudan, Somalia, and Kenya) are affected the most (*2*,*7*). Little is known about the epidemiology and transmission of RVFV in Rwanda; however, high seroprevalence of RVFV was detected in the country during 2012–2013, and an outbreak occurred in 2018 (*8*,*9*). To help fill in knowledge gaps and evidence to guide strategic planning and interventions to prevent RVF outbreaks in Rwanda, we report an epizootic of RVF among livestock in Rwanda that occurred in 2022.

## The Study

In response to a sudden increase in abortion rate among livestock that was reported by animals’ owners in mid-March 2022, Rwanda Biomedical Centre (Kigali, Rwanda), the leading implementer of health systems in the country, initiated an epidemiologic investigation. Initial serologic analysis confirmed exposure of the dead and aborted animals to RVFV. Accordingly, a national health emergency alert was released to engage the community of the animals’ owners, animal health authorities, and community health workers, as well as healthcare providers to enhance collaborative surveillance to strengthen national preparedness, response, and resilience to the health emergency (*10*). The collaborative surveillance included syndromic surveillance implemented through community engagement and supported by molecular epidemiology analysis.

We initially confirmed active RVFV infections by molecular analysis (PCR) on March 22, 2022, in the Nyagatare district in the northeastern region of Rwanda, near the borders with Tanzania and Uganda. However, the epizootic grew and spread rapidly among the populations of livestock throughout the country (Appendix Figure 1). The epizootic peaked at 77 cases reported on April 14, 2022, and disease fatalities peaked at 41 deaths reported on May 26 (Figure). The epizootic ended by October 14, 2022.

**Figure:**
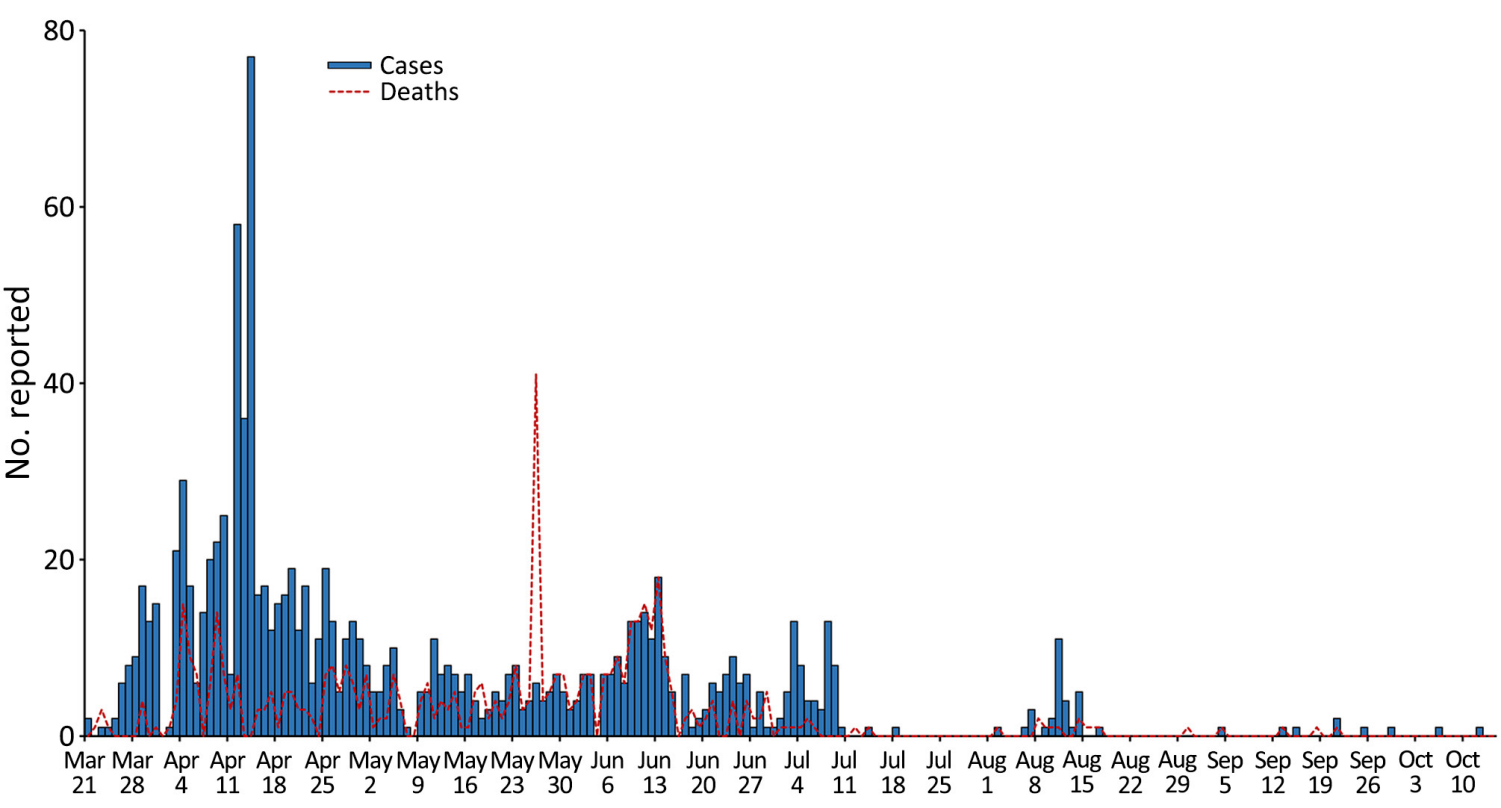
Cases and deaths among livestock, by date reported, during Rift Valley fever epizootic in Rwanda, March 21–October 14, 2022.

During the epizootic, March–October 2022, a total of 3,112 infections were confirmed among livestock, representing 0.2% of the total population of animal resources (livestock) in the country. Confirmed RVFV infections included 1,342 cases, 1,254 abortions, and 516 deaths (Table). Most cases were reported from Eastern 535 (40%) and Southern 450 (33%) Provinces, as were abortions (41% were reported from Eastern and 41% from Southern Provinces). Most fatalities (225 [44%]) were reported from Southern Province, closely followed by Eastern Province (145 [28%]) (Table). Of note, the lowest proportion of RFV cases was in Western Province: 49 (4%) cases, 13 (1%) abortions, and 18 (3%) deaths. Cross-species analysis indicated that most RVF cases (1,285; 96%) affected the bovine population (Table); however, the reporting of abortions and deaths was not disaggregated by animal species.

**Table:** Epidemiologic characteristics of the Rift Valley fever epizootic in Rwanda, 2022

Province	No. animals	Epidemiologic characteristics, no. (%)					Distribution of cases by species		
		Cases	Abortions	Fatalities	Affected animals		Bovine	Caprine	Ovine
Central	73,539	99 (7)	122 (10)	45 (9)	266 (9)		96	1	2
Eastern	1,171,793	535 (40)	511 (41)	145 (28)	1,191 (38)		532	0	3
Northern	635,259	209 (16)	87 (7)	83 (16)	379 (12)		178	18	13
Southern	911,211	450 (33)	521 (41)	225 (44)	1,196 (38)		435	11	4
Western	625,256	49 (4)	13 (1)	18 (3)	80 (3)		44	4	1
Total	1,880,591	1,342 (100)	1,254 (100)	516 (100)	3,112 (100)		1,285	34	23

Although cases of RVF in cattle were distributed throughout the country, the heavy burden was reported from the central and southern regions of Rwanda (Appendix Figure 2). Cases of RVF in goats were scattered throughout the northern and southwestern regions of the country. Cases of RVF in sheep were distributed from northern to southern Rwanda. However, it was not possible to investigate the drivers behind the sudden emergence and spread of the outbreak (*1*,*6*). 

The capacity of entomologic surveillance and response in Rwanda is limited. Therefore, no entomologic investigations were performed to identify the vector species involved in the epizootic.

## Conclusions

The emergence of the countrywide RVF epizootic in Rwanda suggests changes in disease transmission in the country, which could be attributed to increased density and mobility of livestock and to changes in vector composition resulting from emergence of invasive disease vectors (*11*). Because no entomologic investigations were undertaken during the epizootic, information about vector species involved in the outbreak, as well as the presence and distribution of RVF-competent vectors, is not available. The potential change in the composition of vectors might have been influenced by the recent expansion of rice farming in the country. Nevertheless, considering the growing risk for invasive-disease vectors and the growing burden of vector-borne diseases in the region, more investment should be made in building technical expertise and capacity to routinely implement comprehensive vector surveillance and control, with a focus on early detection of invasive vectors (*11*,*12*). Raising awareness and engaging the community in implementing syndromic surveillance will help with early detection and response (*10*). However, further investigations are needed to understand the driving factors behind the development and spread of RVF outbreaks (*13*).

To shed some light on the cross-border dynamics of RVFV in Rwanda, further genomic investigations are warranted (*14*) and should generate evidence that helps strengthen implementation of the International Health Regulations (2005) (https://www.who.int/publications/i/item/9789241580496) to prevent and control cross-country transmission of diseases including RVF (*6*,*15*). However, RVF is on lists of the World Health Organization, GAVI (https://www.gavi.org), and the Coalition for Epidemic Preparedness Innovations (https://cepi.net) for “disease X” pathogens and for pandemic-prone diseases. Therefore, stakeholders of human, animal, and environment health in Rwanda should prioritize strengthening the local pandemic preparedness, prevention, and response framework through a multisectoral transdisciplinary One Health strategy (*1*,*15*).

Widespread RVF infections among livestock in Rwanda suggest that the disease is endemic to the country and that factors such as increased density and mobility of livestock and changes in climate or vector composition might have enhanced transmission. Therefore, a strategy of strengthening the pandemic preparedness, prevention, and response framework in the country, including community-based syndromic surveillance, would be helpful. Because of the wide range of hosts susceptible to RVF, the framework should incorporate a multisectoral transdisciplinary One Health strategy to effectively protect humans, animals, and the environment from the devastating health, safety, food insecurity, and socioeconomic effects of RVF outbreaks.

Appendix Additional information for Rift Valley fever epizootic, Rwanda, 2022.
