# Supplementary material for: Rift Valley Fever Epizootic, Rwanda, 2022
Source: Emerg Infect Dis. 2024 Oct;30(10):2191–3. doi: 10.3201/eid3010.240264 (PMC11431898; doi:10.3201/eid3010.240264)
Supplement: Appendix — Additional information for Rift Valley fever epizootic, Rwanda, 2022. [file 24-0264-Techapp-s1.pdf]

Article DOI: https://doi.org/10.3201/eid3010.240264

# Rift Valley Fever Epizootic, Rwanda, 2022

## Appendix

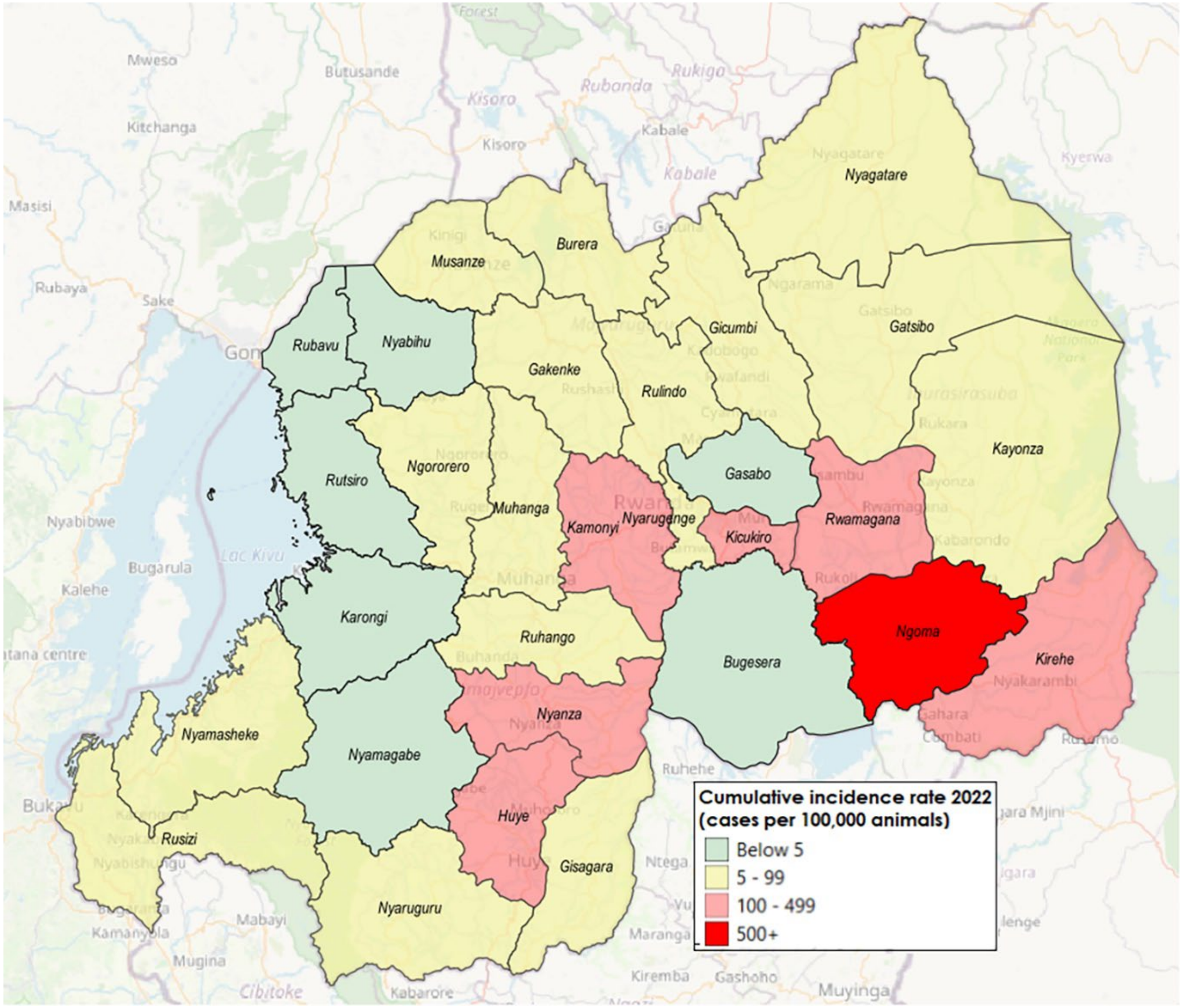


**Appendix Figure 1**. Geographic distribution of Rift Valley fever infections among livestock in Rwanda, March 21–October 14, 2022.

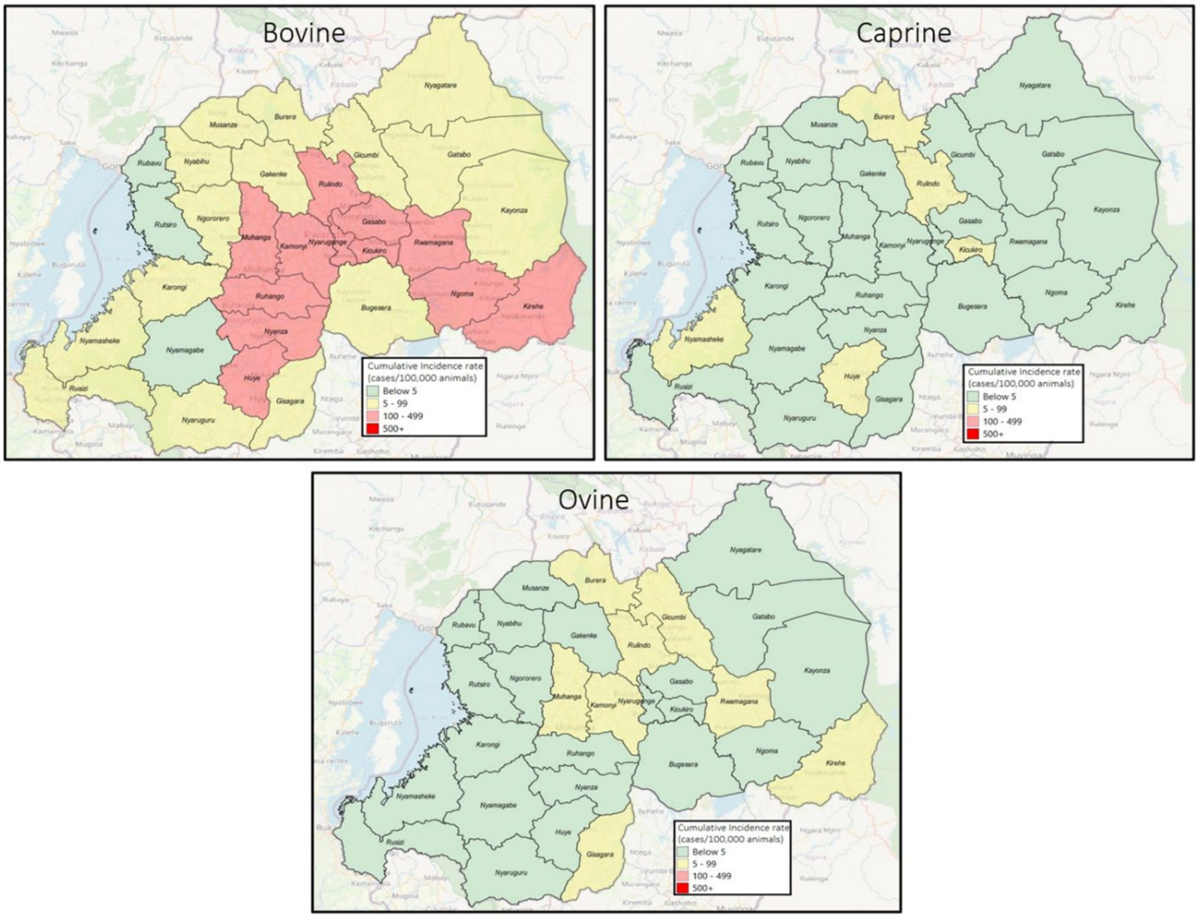


**Appendix Figure 2.** Geographic distribution of cases, by livestock species type, during Rift Valley fever epizootic in Rwanda, March 21–October 14, 2022.
